# A Simple and Effective Approach Based on a Multi-Level Feature Selection for Automated Parkinson’s Disease Detection

**DOI:** 10.3390/jpm12010055

**Published:** 2022-01-06

**Authors:** Fatih Demir, Kamran Siddique, Mohammed Alswaitti, Kursat Demir, Abdulkadir Sengur

**Affiliations:** 1 Biomedical Department, Vocational School of Technical Sciences, Firat University, Elazig 23000, Turkey; fatihdemir@firat.edu.tr; 2 Department of Information and Communication Technology, School of Computing and Data Science, Xiamen University Malaysia, Sepang 43900, Malaysia; 3 Department of Information and Communication Technology, School of Electrical and Computer Engineering, Xiamen University Malaysia, Sepang 43900, Malaysia; 4 Mechatronics Engineering Department, Technology Faculty, Firat University, Elazig 23000, Turkey; kursatdemir62@gmail.com; 5 Electrical-Electronics Engineering Department, Technology Faculty, Firat University, Elazig 23000, Turkey; ksengur@firat.edu.tr

**Keywords:** Parkinson’s disease, multi-level feature selection, optimized KNN

## Abstract

Parkinson’s disease (PD), which is a slowly progressing neurodegenerative disorder, negatively affects people’s daily lives. Early diagnosis is of great importance to minimize the effects of PD. One of the most important symptoms in the early diagnosis of PD disease is the monotony and distortion of speech. Artificial intelligence-based approaches can help specialists and physicians to automatically detect these disorders. In this study, a new and powerful approach based on multi-level feature selection was proposed to detect PD from features containing voice recordings of already-diagnosed cases. At the first level, feature selection was performed with the Chi-square and L1-Norm SVM algorithms (CLS). Then, the features that were extracted from these algorithms were combined to increase the representation power of the samples. At the last level, those samples that were highly distinctive from the combined feature set were selected with feature importance weights using the ReliefF algorithm. In the classification stage, popular classifiers such as KNN, SVM, and DT were used for machine learning, and the best performance was achieved with the KNN classifier. Moreover, the hyperparameters of the KNN classifier were selected with the Bayesian optimization algorithm, and the performance of the proposed approach was further improved. The proposed approach was evaluated using a 10-fold cross-validation technique on a dataset containing PD and normal classes, and a classification accuracy of 95.4% was achieved.

## 1. Introduction

Parkinson’s disease (PD) is defined as a kind of progressive and neurological disorder that causes permanent cessation of brain cells and that is generally diagnosed at ages of above 60 years [1]. If PD is diagnosed at an early stage, the progress of the disease is significantly slowed down with appropriate treatment methods. PD has motor and non-motor symptoms, such as vocal disorders, tremors, slowness, visceral/musculoskeletal pain, stiffness, sadness, increased anxiety, pessimism, and loss of pleasure at early stages [2]. Since the vocal disorders in PD are the most significant symptom for early diagnosis, specialists have focused on abnormalities in voice characteristics, such as loss of freshness, color, and strength of volume [3,4]. The diagnosis of PD generally includes experiments, invasive methods, and empirical tests that have reliability problems. Moreover, these methods are neither cost-effective nor practical due to the use of mixed equipment structures. Additionally, specialists may make a wrong decision due to absent-mindedness or workload as a result of so many transactions [5]. For these reasons, the detection of early stage PD using machine learning methods from voice signals is a turning point to prevent redundant visitations to clinics, decrease the caseload of doctors, and increase the possibility of controlling the illness to achieve recovery and treatment. Moreover, these approaches are cost-effective, simple, and more accurate [6].

The use of machine learning techniques for the automated detection of PD consists of certain general stages. First, to obtain clinically helpful data, different pre-processing algorithms are applied to speech signals. Second, the features are extracted and conveyed to different classification algorithms. Therefore, the selection of classification algorithms and feature extraction processes substantially affects the certainty and dependableness of the proposed system [7]. 

In this study, a new and effective approach based on multi-level feature selection called CLS was proposed to automatically detect PD using voice-based features that had been extracted from voice records of PD cases. CLS feature selection increased the performance of the proposed method. The optimized KNN was used in the classification process. The contribution of the proposed method can be expressed as follows:

− Compared to popular deep learning models, the computational cost of the proposed approach is very low. Therefore, the proposed approach can be applied in clinical practice with low-capacity hardware.
− By using CLS feature selection, higher performance was achieved with fewer features.
− Due to the small number of iterations, the hyperparameters that achieved the best performance in the KNN classifier were found with the Bayesian algorithm.

## 2. Literature Work

When the related literature was investigated, it was seen that the research could be divided into two parts, namely feature-based PD detection methods and classifier-based PD detection methods. 

For instance, Sakar et al. [8] used the machine learning-based classification approach for samples of voices containing words, sustained vowels, and sentences obtained from speaking samples of PD cases. They concluded that sustained vowels gave more discriminative and effective results in comparison to short sentences and words. The s-LOO validation methods and Leave-One-Subject-Out (LOSO) were used to evaluate the achievement of the KNN and Lib-SVM classifiers. Vásquez-Correa et al. [9] studied voice recordings of three languages, Spanish, German, and Czech. To distinguish voiced segments from unvoiced ones, speech signals were classified using articulation. Then, speech signals with time-frequency components in transition were analyzed and reached an accuracy rate up to 89% in the patient and healthy case classification problem that was presented in the study. Goberman [10] investigated the relationship between different movement and speech features to determine cases of PD by examining the motor performance with the Unified Parkinson’s Disease Rating Scale and speech through the acoustic analysis of prosody, articulation, and phonation. It was concluded that movement features (posture, facial expression, gait, postural stability, rest tremor, and postural tremor) were significantly related to 7 acoustic measures of 16 speech features. Little et al. [11] proposed two different tools, fractal and recurrence scaling, for speech analysis. These scaling types, which provided a diagram of dysphonia by considering two symptoms of disease, eliminated the range constraints of other tools. Thus, these two characteristics were used to differentiate healthy subjects from others. The classification performance achieved score of 95.4% with a 2.0% error margin and 91.5% with a 2.3% error margin, respectively (%95 confidence). Rusz et al. [12] evaluated the detection of speech disorders at early stages through vocal deformations for the classification of PD patients and healthy people. They proposed that distinctions occurring in the fundamental frequency were the main reasons that 78% of the PD cases at early phases had vocal deformations. Tsanas et al. [13] utilized an updated dataset comprising 43 subjects 263 speech samples. While 23.3% of the dataset comprised samples from healthy subjects, the rest comprised samples from PD patients. The study achieved approximately 99% classification accuracy by utilizing 10 hoarseness attributes. 

Gök [14] conducted an ensemble of k-nearest neighbor (k-NN) algorithms to improve classification performance. The model of a chosen feature subset was conducted to different classifiers to detect illness, and a 98.46% accuracy rate was obtained. Bayestehtashk et al. [15] studied speech signals in order to grade and detect PD severity through the use of sustained phonations, a system with regression analysis, to estimate PD severity. Taha et al. [16] investigated how to categorize speech signals using the SVM classifier and the 10-fold cross-validation method. The 20 healthy and 60 patient subjects were examined, their 240 recorded running voice samples were used to create a dataset. The unified Parkinson’s score scale motor exam of speech (UPDRS-S) was used to clinically rate these speech samples. Wen et al. [17] studied an effective classification and feature selection approach based on RBF-SVM and a Haar-like feature selection method. Cantürk and Karabibe [18] extracted features by using the MRMR, RELİEF, LLBS, and LASSO algorithms from speech signals. SVM, Naïve Bayes (NB), Multilayer Perceptron (MLP), k-NN, and Ada boost classifiers were utilized to separate PD patients from healthy people. Cai et al. [19] utilized an algorithm containing relief feature selection and SVM classifiers for detecting PD. They utilized BFO, bacterial foraging optimization, to improve classification performance. S. Ashour et al. [20] presented a method based on two-level feature selection for PD detection. At the first level, two feature sets were constituted with the eigenvector centrality feature selection (ECFS) and the principal component analysis (PCA) algorithms. At the second level, weighted feature selection was used because the ECFS method provided better performance than PCA. In the classification stage, the SVM classifier reached a classification accuracy of 94%. Haq et al. [21] used L1-Norm SVM feature selection to obtain distinctive features from voice signals from PD voice samples. The selected features were classified with the SVM algorithm. The 10-fold cross-validation technique was utilized to evaluate the proposed system.

## 3. Dataset

Audio signals obtained from 252 cases (188 PD and 64 healthy) in a group of volunteers were used to create the dataset [22]. Audio signals were collected three times from all cases, and the dataset was increased to 756 samples containing 564 PD classes and 192 Healthy classes. The vocal features of softening, monotonous, brittle, and rapid expression were constituted with these samples. To determine a speech disorder in the PD cases, the voice-based features were extracted from each of the samples in the dataset. A total of 21 features were extracted from baseline methods and contained harmonicity and fundamental frequency parameters. Wavelet transform (WT), time-frequency (TF), tunable Q-factor wavelet transform (TQWT), Mel frequency Cepstral coefficients (MFCCs), and vocal fold (VF) algorithms were used to extract the other 732 features.

## 4. Methodology and Machine Learning Techniques

### 4.1. Methodology

**Figure 1:**
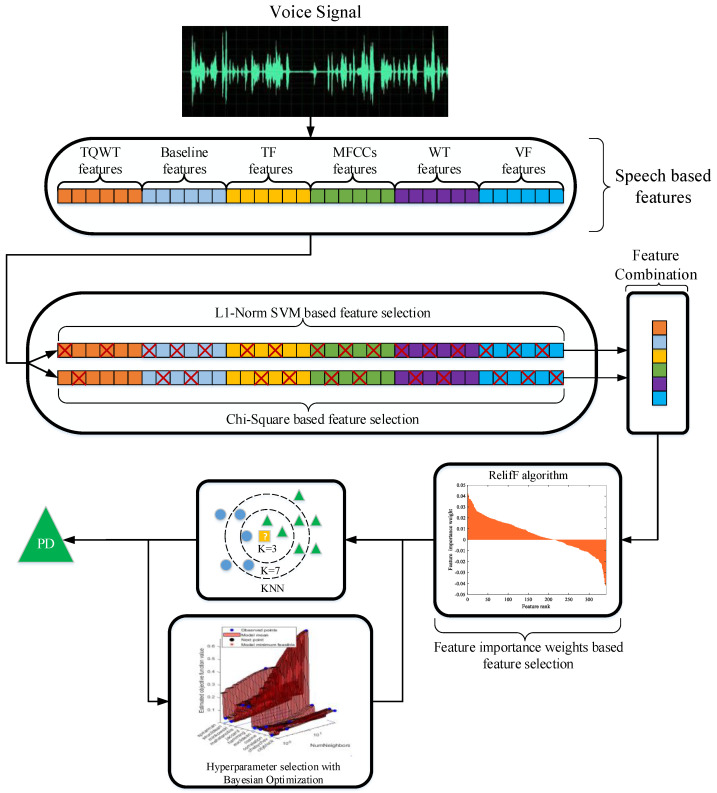
The illustration of the proposed approach.

The illustration of the proposed approach based on multi-level feature selection, called CLS, is given in Figure 1. The dataset that was used was composed of the voice-based features that had been extracted from sounds taken from the PD cases. The proposed method was composed of a 3-level feature selection process for improving the classification performance. At the first level, two feature sets were constituted with the Chi-square algorithm and the L1-Norm SVM. In the Chi-square algorithm, the number of features to be selected was adaptively determined according to the classification error. In the L1-Norm SVM algorithm, the number of features to be selected was determined based on punishment parameter *C*. Two feature sets were concatenated at the second level. At the third level, the distinctive features were selected using a feature importance weights-based approach using the ReliefF algorithm from the concatenated feature set. The steps of the CLS feature selection approach are as follows.

Step 1: Load features.
Step 2: Adaptively create the first feature set according to classification error with the Chi-square algorithm.
Step 3: Constitute the second feature set according to the punishment parameter (*C*) with the L1-Norm SVM algorithm.
Step 4: Concatenate each two feature sets.
Step 5: Compute the feature importance weights using the ReliefF algorithm.
Step 6: Remove negative weights in the calculated weights from the concatenated feature set.

The KNN classifier was used in the classification stage since it provided higher classification performance compared to other popular classifiers such as SVM, Decision Tree (DT), and Naïve Bayes (NB). Moreover, the hyperparameters of the KNN were optimized with the Bayesian algorithm for increasing the success rate of the proposed approach. Accuracy, which was the main criterion, sensitivity, specificity, precision, and F-score metrics were used to evaluate the performance of the proposed approach.

### 4.2. Multi-Level Feature Selection

The reason for using a multi-level feature selection approach is that no single algorithm can achieve the best performance in feature selection. For example, when there is an outlier in the attribute data, thresholding-based feature selection algorithms remove features that are important for classification. Correlation-based feature selection algorithms are good at finding outliers, but features with the same local characteristics can be removed [23,24]. Therefore, using both types of feature algorithms can improve classification performance. In this study, the L1-norm SVM algorithm was used for the statistical and threshold-based feature selection algorithm since it provided high classification performance in many existing methods [25,26,27]. The Chi-square is the most used correlation-based feature selection algorithm [28,29,30,31,32,33]. The features that were obtained from both algorithms were stacked in a vector. To further reduce the computational cost without sacrificing performance, the features in this vector were selected by the ReliefF algorithm according to their weight values.

### 4.3. L1-Norm SVM Algorithm

The number of features was determined according to the cost parameter for the feature selection-based L1-Norm SVM [21]. The dataset with *n* samples is expressed in the equation below:

(1) $$S={\left\{\left(x_{i},y_{i}\right)|x_{i}\in R^{n},y_{i}\in \left\{-1,\;1\right\}\right\}}_{i=1}^{k}$$

where $x_{i}$ is the ith sample which has *n* features and a class label ($y_{i}$). 

The SVM in the classification problem with two classes (Equation (3)) learns the separating hyper-plane that makes margin size maximum.

(2) $$y_{i}\left(wx_{i}-b\right)\geq 1,\;i=1,\ldots ,k$$

where the bias term and weight vector are w and *b*, respectively. The optimization problem that was determined in Equation (4) based on the problem in Equation (3) needs to be solved.

(3) $$\min\frac{1}{2}{\left\|w\right\|}^{2}$$

The formulation determined in Equation (3) can be re-arranged as Equation (4) to correct classification errors resulting from the distance from the margin.

(4) $$y_{i}\left(wx_{i}-b\right)\geq 1-\delta ,\;\delta i\geq 0,i=1,\ldots ,k$$

Bradley and Mangasarian [34] used Equation (3) by accepting Equation (5) as a constraint for the feature selection-based L1-Norm SVM as a result of sparse solutions.

(5) $${\min\|w\|}^{1}+C\overset{k}{\underset{i=1}{\sum }}\max{\left(0.1-y_{i}\left(\alpha^{T}x_{i}+b\right)\right)}^{2}$$

where α, the Lagrange [35] is the weight vector obtained from the optimization multiplier. Moreover, the value of the *C* parameter used in Equation (5) determines the size of the feature set.

### 4.4. Chi-Square Algorithm

In the chi-square algorithm, a feature set $t_{i}$ is chosen according to its correlation with a $C_{j}$ class, and the discriminating ability of feature $t_{i}$ following $C_{j}$ class is calculated as below:

(6) $$x^{2}\left(t_{i},C_{j}\right)=\frac{N\times {\left(a_{ij}d_{ij}-b_{ij}c_{ij}\right)}^{2}}{\left(a_{ij}+b_{ij}\right)\times \left(a_{ij}+c_{ij}\right)\times \left(b_{ij}+c_{ij}\right)\times \left(c_{ij}+d_{ij}\right)}$$

where N is the number of total samples. $a_{ij}$ is the number of samples containing feature $t_{i}$ in the $C_{j}$ class, and $b_{ij}$ is the number of samples not containing feature $t_{i}$ in the $C_{j}$ class [36]. $c_{ij}$ is the number of samples with feature $t_{i}$ that is not in the $C_{j}$ class. Lastly, $d_{ij}$ is the number of samples with neither feature $t_{i}$ nor the $C_{j}$ class.

### 4.5. ReliefF Algorithm

The ReliefF algorithm computes the predictor weights if the target classes are multi-class categorical values. The predictors giving different scores to neighbors in the same class are penalized, while the predictors giving the same scores to neighbors in the same class are rewarded [37]. In the ReliefF algorithm, all predictor weights (*W_j_*) are first set to 0. Then, the ReliefF algorithm recurrently chooses a random prediction (*x_s_*), calculates the k-nearest predictions to *x_s_* in each class, and updates according to each nearest neighbor (*x_t_*) [38,39]. If the classes of *x_s_* and *x_t_* are the same, then all of the weights for the predictors (*P_i_*) are as follows:

(7) $$W_{i}^{j}=W_{i}^{j-1}-\frac{\Delta_{i}\left(x_{s},x_{t}\right)}{n}d_{st}$$

If the classes of *x_s_* and *x_t_* are different, then all the weights for the predictors (*P_i_*) are as follows:

(8) $$W_{i}^{j}=W_{i}^{j-1}-\frac{p_{y_{s}}}{1-p_{y_{t}}}\cdot \frac{\Delta_{i}\left(x_{s},x_{t}\right)}{n}d_{st}$$

where $W_{i}^{j}$ denotes the weight of the *P_i_* for the *j*th iteration, $p_{y_{s}}$ represents the previous possibility of the class to which *x_s_* belongs, $p_{y_{t}}$ represents the previous possibility of the class to which *x_q_* belongs, *n* is the number of iterations tuned by updates, and Δ*i*(*x_s_*, *x_t_*) is the difference in the score of the predictor *P_j_* between observations *x_s_* and *x_t_*. For discreteness, the *P_i_*, Δ*i*(*x_s_*, *x_t_*) can be expressed as follows:

(9) $$\Delta i\left(x_{s},x_{t}\right)=\{\begin{array}{cc} 0, & x_{s\left(i\right)}=x_{q\left(i\right)}\\ 1 & x_{s\left(i\right)}\neq x_{q\left(i\right)} \end{array}$$

the distance function ($d_{st}$) and the distance function ($d_{st}^{\sim }$) are stated as follows:

(10) $$d_{st}=\frac{d_{st}^{\sim }}{\sum_{l=1}^{L}d_{sl}^{\sim }}$$

(11) $$d_{st}^{\sim }=e^{-{\left(rank\left(s,t\right)/sigma\right)}^{2}}$$

where *rank*(*s*,*t*) is the location of the *t*th observation between the nearest neighbors of the *s*th observation, which is ranked by distance. *L* is the number of nearest neighbors, which is represented by *L*.

### 4.6. KNN Classifier

In addition to the use of the k-Nearest Neighbors (KNN) algorithm for both classifier and regression problems in the supervised learning category, it is also preferred to solve classifying problems for application-based practice [40]. Cover and Hart [41] proposed a dataset comprising predetermined labels in the KNN algorithm. According to the nearest neighbors, the new data to be classified in the KNN algorithm are categorized from a labeled dataset. The distance and data in the labeled dataset are used to determine the class of new data [42]. These distances are computed with a distance metric such as the Euclidian, Minkowski, Chebychev, and Manhattan metrics.

### 4.7. Bayesian Optimization

The achievement of methods is substantially related to the hyperparameters that are chosen automatically or manually in machine learning algorithms. Manual selections of the hyperparameters need experts and also have the possibility of failing to obtain optimal conclusions on the first try. Moreover, running the algorithms several times may be required for the fine adjustment of the hyperparameters [43]. Although the grid search and random search-based optimization algorithms are usually applied to obtain optimum hyperparameters, the solution of the optimization problem using these algorithms needs a timewasting operation in deep learning models with big data. The Bayesian optimization algorithm is an effective method that can be used to figure out functions with better computational costs [44]. The optimization target to reach the global maximum value in a black-box function is calculated in Equation (12):

(12) $$x^{-}=argmax_{x\in S}f\left(x\right)$$

where S is the searching in *x*. For evidence data D in the Bayesian theorem, the posterior probability P(E|D) of pattern E is computed in Equation (13):

(13) P(E|D )=P(D|E)P(E)

where P(E) is the former probability, and P(D|E) is the possibility of the learning data D. The Bayesian optimization algorithm provides the combination of the foregoing distribution of the function f(x) with the instances of the previous information to obtain the posteriors. The maximization value of the f(x) is described by the posteriors computing the validation, and the utility function (p) is the maximization term. The phases of the Bayesian optimization algorithm, including the training data (T) and the observation numbers (*n*), are stated below:

− Find $x_{n}$ by optimizing the utility function p with a specific iteration→ $\begin{array}{c} x_{n}=argmax\;p(x|T_{1:n-1})\\ x \end{array}$
− Examine the objective function → $y_{n}=f\left(x_{n}\right)$
− Put new values and update the data → $T_{1:n}=\left\{T_{1:n-1},\left(y_{n},x_{n}\right)\right\}$

## 5. Experimental Studies

**Figure 2:**
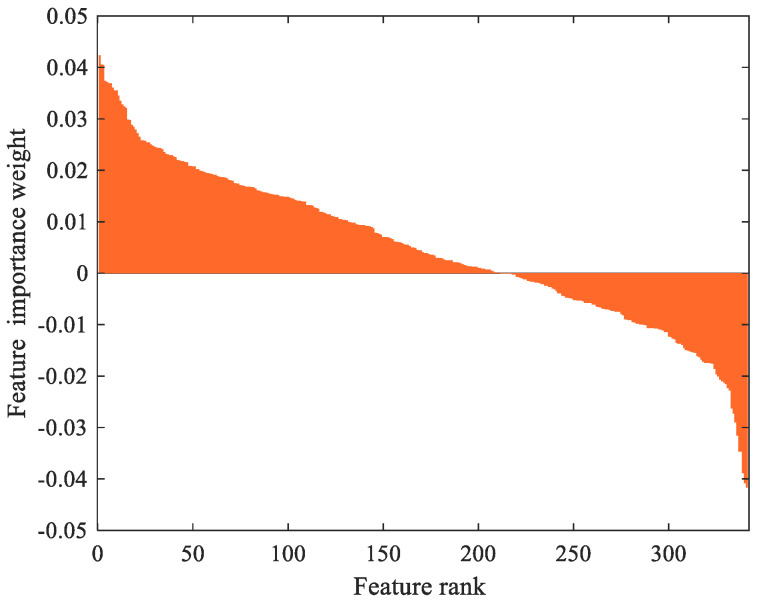
Feature importance weights of the concatenated features.

In this study, the coding processes were performed with MATLAB 2019a and Python 3.6 programs installed on hardware that had an Intel^®^ Core™ i7-5500U CPU with a 2.4 GHz graphics card with 2GB random access memory with 8GB DDR3, and a Windows 10 operating system. In the Chi-square algorithm, the number of features, which was set to 300, was adaptively selected with regard to the minimum classification error. In the L1-Norm SVM algorithm, the punishment parameter *C* was set to 0.01, and 41 distinctive features were selected by this parameter. A total of 341 features was achieved by concatenating two feature sets. Using the ReliefF algorithm, 341 features were reduced to 220 features, and features with positive importance weights were calculated as the predictors. In Figure 2, positive and negative feature importance weights are given according to the feature rank. As seen in Figure 2, after 220 features, the feature importance weights became negative. Therefore, the features containing negative importance weights were removed from the concatenated features set.

**Figure 3:**
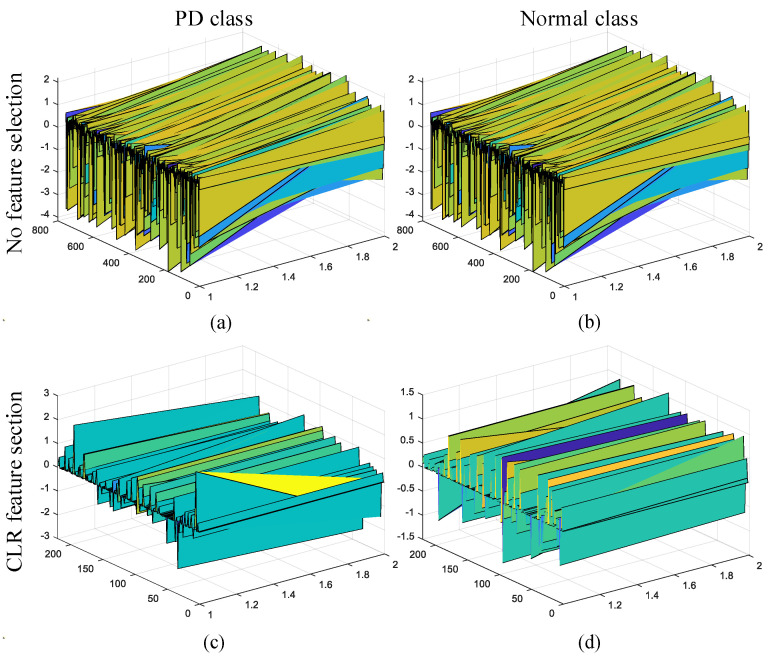
3D feature representations for PD and normal classes according to feature selection cases: (**a**) the feature representation for PD class and no feature selection; (**b**) the feature representation for Normal class and no feature selection; (**c**) the feature representation for PD class and CLS feature selection; (**d**) the feature representation for normal class and CLS feature selection.

In Figure 3, the 3D representation of 756 features without feature selection operation and on 220 features with the CLS feature selection are shown for the PD class and the normal class. As seen in Figure 3, the distinction between the two classes was clear by the CLS feature selection algorithm, and the computational cost was reduced.

The DT, Linear Discriminant (LD), NB, SVM, and KNN algorithms were utilized in the classification stage. For the DT classifier, the best performance was achieved with the medium DT, where the maximum number of splits and split criterion parameters were chosen as 20, and the “Gini diversity index”. For the LD classifier, the best performance was obtained by denoting the covariance structure parameter as being full. The Gaussian kernel was used for the NB classifier. The linear, polynomial, radial basis function (RBF), and Gaussian kernels were utilized in the SVM classifier. The best accuracy was achieved with the SVM with the polynomial kernel. In the KNN classifier, the highest accuracy was achieved with the Fine KNN classifier, wherein the number of nearest neighbors, the distance metric, and the distance weight were “1”, “Euclidian”, and “Equal”, respectively. 

In Table 1, the classification scores of DT, LD, NB, SVM, and KNN are given for each of the Chi-square, the L1-Norm SVM, and the ReliefF algorithms. To objectively compare the results in Table 2, 220 features were selected using each feature selection algorithm. As seen in Table 1, when each feature selection algorithm was used alone, the classification performance was improved for almost all classifiers. As seen in Table 1, the best and worst performances for all of the classifiers were obtained with the ReliefF and Chi-square algorithms, respectively.

In Table 2, the accuracy performances of DT, LD, NB, SVM, and KNN are given for three different situations, which included 752 features (no feature selection), 341 features (the concatenated features with the Chi-square and the L1-Norm SVM algorithms), and 220 features (the CLS feature selection). The evaluation was performed with the 10-fold cross-validation. For all classifiers, the best accuracies were reached with the CLS feature selection algorithm, and the worst accuracies were achieved without feature selection operation. As seen in Table 2, the best accuracy was obtained with the KNN (fine) classifier for all situations. In the KNN classifier, the accuracy performance was improved by 2% with the concatenated features and by 4.6% with CLS feature selection. Among all of the classifiers, the performance of the LD classifier was shown to be the most improved CLS feature selection was used.

To improve the classification performance of the KNN, the hyperparameters containing the distance metric, the number of neighbors, and the distance weight were optimized with the Bayesian algorithm. The distance metric, the number of neighbors, and the distance weight were searched between the options and values given in Table 3.

**Figure 4:**
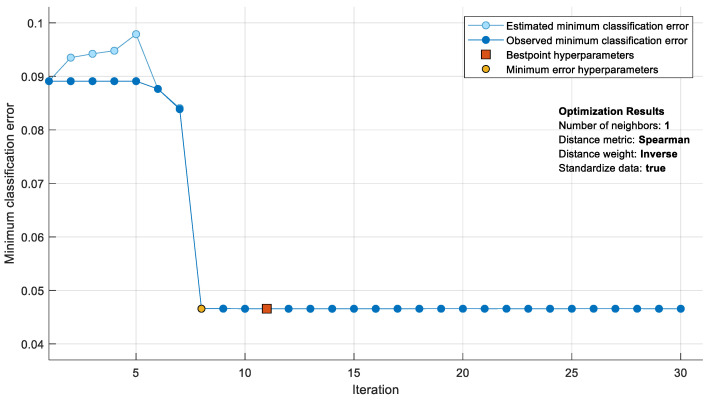
Change of minimum classification error in the KNN during Bayesian hyperparameter optimization.

In Figure 4, the minimum classification error values of the KNN are given during a Bayesian optimization process with 30 iterations. At the end of 30 iterations, the best minimum classification error was obtained when the Spearman coefficient for the distance metric was determined to be “1” for the number of neighbors when the inverse coefficient for the distance weight and was 0.046. The hyperparameters providing the minimum classification error value were achieved between the 10th and 15th iterations.

**Figure 5:**
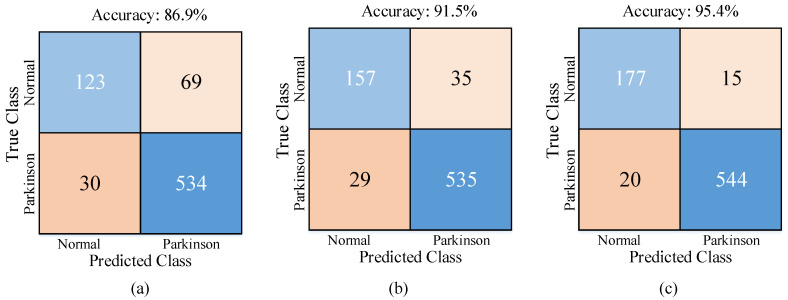
Confusion matrices for three different cases: (**a**) The Fine KNN without feature selection; (**b**) The Fine KNN with the CLS feature selection; (**c**) The optimized KNN with the CLS feature selection.

**Figure 6:**
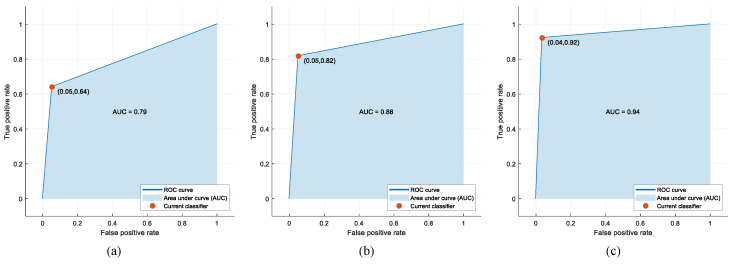
ROC curves and AUC values for three different cases: (**a**) The Fine KNN without feature selection; (**b**) The Fine KNN with the CLS feature selection; (**c**) The optimized KNN with the CLS feature selection.

For 3 different cases of the Fine KNN without feature selection, the Fine KNN with CLS feature selection, and the optimized KNN with CLS feature selection, the confusion matrices, the other performance metric results, and the ROC curves and AUC values are given in Figure 5, Table 4, and Figure 6, respectively. 

As seen in Figure 5, the best true positive (TP) and true negative values were achieved with the optimized KNN using CLS feature selection (Figure 5c). Compared to the Fine KNN without feature selection, the accuracy score was improved by 4.6% CLS feature selection was used and by 8.5% with CLS feature selection and the Bayesian hyperparameter optimization were used.

As seen in Table 4, with the optimized KNN and the CLS feature selection, the best sensitivity was 0.96 for the PD class, the best specificity was o 0.96 for the normal class, the best precision was 0.97 for the PD class, and the best F-score was 0.96 for the PD class.

As seen in Figure 6, the AUC value was 0.79 for the Fine KNN without feature selection, 0.88 for the Fine KNN with CLS feature selection, and 0.94 for the optimized KNN with CLS feature selection. Compared to the Fine KNN without feature selection, the AUC value was improved by 0.09 with Fine KNN plus CLS feature selection, and a rate of 0.15 was obtained with the optimized KNN plus CLS feature selection.

In Table 5, the obtained scores for the proposed approach were compared to the existing methods using the same dataset. In the baseline method [22], the voice-based features (752 features) were decreased to 50 features with the minimum redundancy-maximum relevance (mRMR) feature selection method. The selected features were trained with an SVM classifier with an RBF kernel. The best accuracy and F-score were 86% and 0.84, respectively. In [20], the two-level feature selection method was applied to the voice-based features. In the first level, the distinctive features were selected with the ECFS and PCA algorithms. In the second level, the selected features were reduced by performing the second application of the ECFS algorithm. An SVM classifier was used to evaluate the method. The best accuracy, sensitivity, specificity, precision, and F-score values were 93.80%, 0.84, 0.97, 0.915, and 0.875, respectively. The proposed approach outperformed these two methods with regard to the used metrics, excluding the specificity. In [45], a novel feature mapping and convolutional LSTM method was used for PD detection. The authors obtained an accuracy score of 94.27% in their work. 

## 6. Discussion

We carried out further experiments where only one sample was used from each subject. Thus, the total number of samples became 252. The evaluation was also performed with the 10-fold cross-validation. Table 6 shows the obtained results.

As seen in Table 6, the calculated accuracy, sensitivity, specificity, precision, and F-score values were 0.917, 0.87, 0.94, 0.913, and 0.918, respectively. When comparing the results from Table 6 to the previous results, it was seen that the decrease in the number of samples yielded a decrease in the performance.

As the dataset was imbalanced, an oversampling method called SMOTE was used for the performance evaluation [46]. The details of the SMOTE method can be seen in [46]. The number of healthy samples increased to alleviate the class imbalance problem, and the obtained results are given Table 7. From Table 7, it was observed that after alleviating the class imbalance problem, the proposed method produced improved results.

## 7. Conclusions

Parkinson’s patients suffer from a variety of symptoms in various parts of the body, including in their speech, which leads to a loss of voice. Many studies based on machine learning approaches have been conducted to find the relationship between speech disorders and PD to further improve the detection and classification of PD cases. In this study, a novel approach based on a multi-level feature selection method called CLS was used to classify normal and PD cases from voice-based features. The classification performances of all of the classifiers used were improved with CLS feature selection. The KNN classifier with an accuracy of 91.5% provided the best classifier performance. Moreover, the hyperparameters of the KNN were optimized with the Bayesian algorithm. The best accuracy, sensitivity, specificity, precision, and F-score were 95.4, 0.949, 0.930, 0.952, and 0.955%, respectively. Compared to the best existing method when using the same dataset, the accuracy, sensitivity, precision, and F-score metrics were improved by 1.6, 0.109, 0.037, and 0.080%, respectively. However, the specificity score was worse by approximately 0.04%.

With this approach, a useful and highly accurate machine learning model has been created for the early diagnosis of PD. Additionally, the proposed approach does not contain a large number of learnable parameters that are commonly found in deep learning models. Therefore, the proposed approach can be used in clinical practice with a less powerful hardware requirements.

## Figures and Tables

**Table 1:** Accuracy performances for the Chi-square, the L1-Norm SVM, and the ReliefF algorithms.

Classifier		Accuracy (%)		
	Raw Features	L1-Norm SVM	Chi-Square	ReliefF
DT	81.1	82.3	81.2	82.5
LD	72.2	72.7	75.0	74.8
NB	74.6	75.3	74.9	75.6
SVM	85.6	85.8	85.6	86.7
KNN	86.9	87.8	87.7	88.6

**Table 2:** Accuracy performances for three different situations.

Classifier	Accuracy (%)		
	752 Features	341 Features	220 Features (Multi-Level)
DT	81.1	81.5	83.7
LD	72.2	81.0	82.0
NB	74.6	77.4	79.6
SVM	85.6	87.5	89.5
KNN	86.9	88.9	91.5

**Table 3:** Hyperparameter searching range.

Hyperparameters		
Distance Metric	Number of Neighbors	Distance Weight
Cityblock	1–378	equal inverse squared inverse
Chebyshev		
Correlation		
Cosine		
Euclidean		
Hamming		
Jaccard		
Mahalanobis		
Minkowski		
Spearman		

**Table 4:** Other performance metric scores.

Classifier	Class	Sensitivity	Specificity	Precision	F-Score
Fine KNN	Normal	0.64	0.95	0.80	0.71
	PD	0.95	0.64	0.89	0.92
Fine KNN and CLS Feature Selection	Normal	0.82	0.95	0.84	0.83
	PD	0.95	0.82	0.94	0.94
Optimized KNN and CLS Feature Selection	Normal	0.92	0.96	0.90	0.91
	PD	0.96	0.92	0.97	0.97

**Table 5:** Performance comparison of the proposed method with other methods.

Methods	Accuracy (%)	Sensitivity	Specificity	Precision	F-Score
Baseline method [22]	86.00	-	-	-	0.840
Ashour et al. [20]	93.80	0.840	0.970	0.915	0.875
Demir et al. [45]	94.27	0.960	0.960	0.910	0.930
Proposed Approach	95.40	0.949	0.930	0.952	0.955

**Table 6:** Performance of the proposed method with only 252 samples from the dataset.

Methods	Accuracy (%)	Sensitivity	Specificity	Precision	F-Score
Proposed Approach	91.67	0.87	0.94	0.913	0.918

**Table 7:** Performance of the proposed method with oversampling method.

Methods	Accuracy (%)	Sensitivity	Specificity	Precision	F-score
Proposed Approach	94.30	0.96	0.96	0.91	0.93

## Data Availability

The used dataset was a public dataset.
